# Attenuated asthma phenotype in mice with a fetal-like antigen receptor repertoire

**DOI:** 10.1038/s41598-021-93553-6

**Published:** 2021-07-09

**Authors:** Regine Stutz, Christopher Meyer, Elisabeth Kaiser, Sybelle Goedicke-Fritz, Harry W. Schroeder, Robert Bals, Christoph Haertel, Tobias Rogosch, Sebastian Kerzel, Michael Zemlin

**Affiliations:** 1 Department of General Pediatrics and Neonatology, Saarland University Medical School, Homburg, Germany; 2 Department of Pediatrics, Philipps-University Marburg, Marburg, Germany; 3 Department of Medicine, University of Alabama at Birmingham, Birmingham, AL USA; 4 Department of Internal Medicine V-Pulmonology, Allergology and Critical Care Medicine, Saarland University, Saarland University Medical School, Homburg, Germany; 5 Department of Pediatrics, Würzburg University Medical Center, Würzburg, Germany; 6 Department of Pediatric Pneumology and Allergy, University Children’s Hospital Regensburg, Campus St. Hedwig, Regensburg, Germany

**Keywords:** Immunology, Immunogenetics, Immunological disorders, Inflammation, Innate immunity, Immunological disorders

## Abstract

We hypothesized that the scarcity of N-nucleotides might contribute to the inability of the neonate to mount a robust allergic immune response. To test this, we used terminal deoxyribunucleotidyl Transferase deficient (TdT^−/−^) mice, which express “fetal-like” T cell receptor and immunoglobulin repertoires with largely germline-encoded CDR3 regions. Intraperitoneal sensitization was followed by aerosol provocation with either PBS or the allergen OVA in both TdT^−/−^ mice and wild-type mice to develop allergic respiratory inflammation. The effects of this procedure were investigated by lung function test, immunological analysis of serum and brochoalveolar lavage. The local T_H_2 cytokine milieu was significantly attenuated in TdT^−/−^ mice. Within this group, the induction of total IgE levels was also significantly reduced after sensitization. TdT^−/−^ mice showed a tendency toward reduced eosinophilic inflow into the bronchial tubes, which was associated with the elimination of respiratory hyperreactivity. In conclusion, in a murine model of allergic airway inflammation, the expression of fetal-like antigen receptors was associated with potent indications of a reduced ability to mount an asthma phenotype. This underlines the importance of somatically-generated antigen-receptor repertoire diversity in type one allergic immune responses and suggests that the fetus may be protected from allergic responses, at least in part, by controlling N addition.

## Introduction

Allergic asthma is a chronic inflammatory disease of the airways characterized by bronchial hyperreactivity and variable airway obstruction^1,2^.

Allergic sensitization is described as a misled classical affinity-driven immune response associated with an imbalance towards a T_H_2 milieu^3,4^. T_H_2-cell derived cytokines such as interleukin 4 (IL-4) promote B cell isotype switching to immunoglobulin E (IgE) which plays a key role in allergic asthma by acting as a link between an allergen and the mast cell to which the IgE is attached by its constant domain to membrane-bound Fcε receptors^5–7^.

We and others have previously shown that in type 1 allergic responses the third complementarity determining region of the H chain (CDR-H3), which encodes for the center of classic immunoglobulin antigen binding sites, often plays a key role in forming the interface between the allergen and IgE^8–11^. In contrast, in atopic dermatitis the circulating IgE repertoire reflects superantigen like activation^12^.

During fetal life, human and murine T cell receptor (TCR) and immunoglobulin (Ig) repertoires are restricted in diversity due to a reduction or absence of terminal deoxynucleotidyl transferase (TdT) activity in the fetal liver, fetal bone marrow, and fetal thymus; and thus a reduction or absence of N nucleotides within their CDR3 regions^13,14^. After birth, the diversity of both the TCR and Ig repertoires expands as a consequence of the contribution of N nucleotide addition to CDR3^13–19^. The diversification of the adaptive antigen receptor repertoires in utero and after birth parallels the developing ability to respond to a broad range of antigens and to generate robust secondary immune responses, including allergic responses.

Although the classic immunoglobulin antigen binding site forms the Ig core of the allergen–antibody interaction, the importance of qualitative properties of the CDR-H3 region for the development of an allergic phenotype has not yet been conclusively clarified. Previous studies show that gene targeted mouse lines with altered diversity gene loci region exhibit differing allergic phenotypes depending on the hydrophobicity of their CDR-H3 regions^8,20^. A mouse strain expressing predominantly neutral amino acids in CDR-H3 developed a strong allergic phenotype to ovalbumin^17^, whereas a repertoire enriched for positively charged CDR-H3 led to a weakened allergic phenotype. However, to the best of our knowledge the role of N regions on the development of allergic respiratory inflammation has not yet been characterized.

The aim of the present study was to investigate the influence of CDR3 repertoires lacking N nucleotides on the development of the allergic phenotype. We found that in a murine model of allergic airway inflammation terminal deoxynucleotidyl transferase (TdT^−/−^) deficient mice, which express “fetal-like” antigen receptor repertoires without N-nucleotides^21^, give substantial indications to develop an impaired immune response.

## Results

### Induction of total IgE levels after sensitization with OVA is significantly reduced in TdT^−/−^ mice

**Figure 1:**
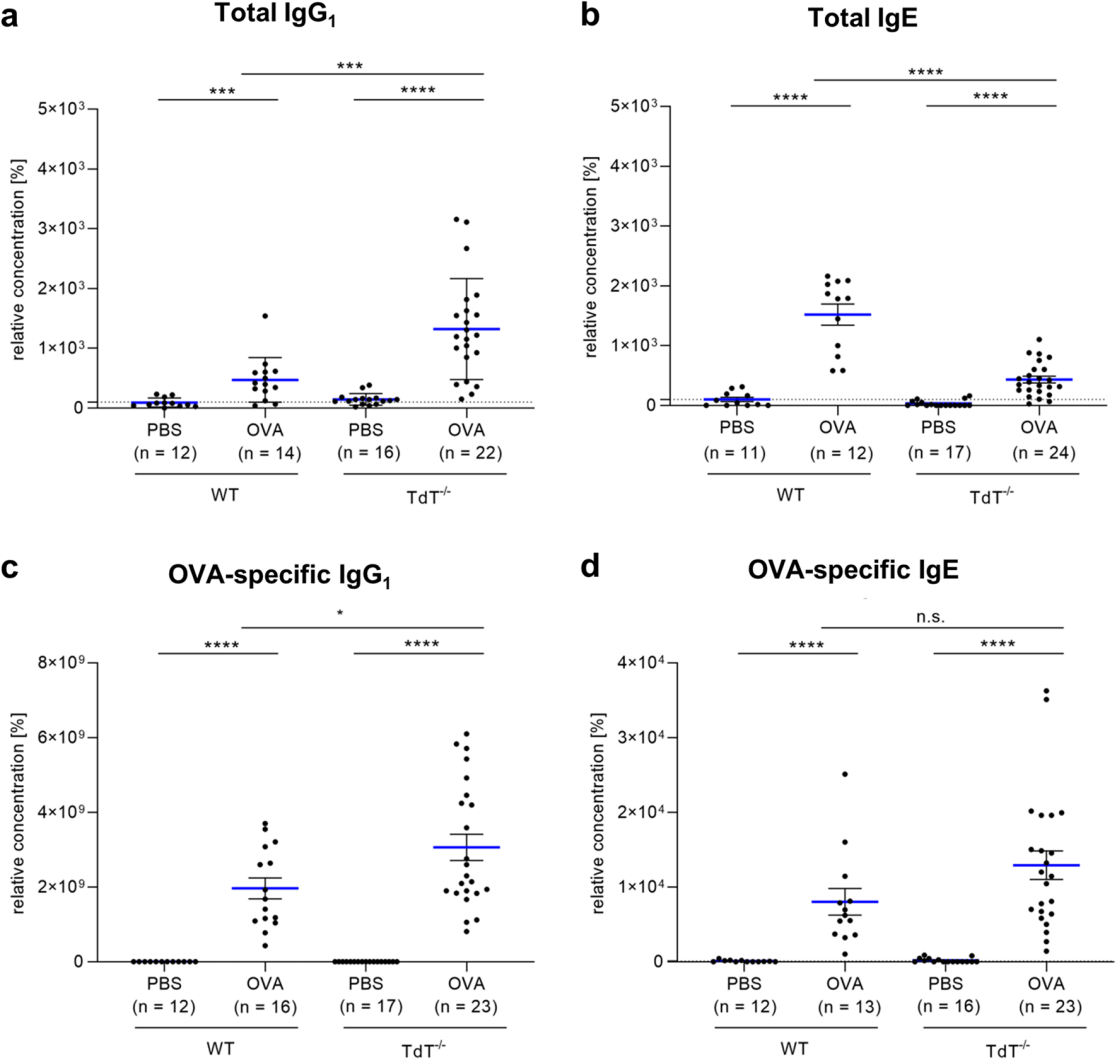
Serum immunoglobulin levels. In serum of *wt* and TdT^−/−^ mice, both sensitized and non-sensitized, immunoglobulin levels of (**a**) total IgG_1_, (**b**) total IgE, (**c**) OVA-specific IgG_1_ and (**d**) OVA-specific IgE were measured using ELISA. Sensitization induced a significant rise in OVA-specific IgG_1_ and IgE levels. No significant differences between *wt* and TdT^−/−^ mice were observed in OVA-specific IgE levels. Total IgE and total IgG_1_ levels were significantly increased after sensitization for both strains. The increase in IgG_1_ levels was significantly higher and the increase in IgE levels was significantly lower in TdT^−/−^ mice when compared to *wt* (mean shown as blue lines, SEM shown as black bars).

To assess allergic sensitization, serum concentrations of IgG_1_ and IgE in the TdT^−/−^ and wt mice were determined. Before sensitization, the serum levels of each of the corresponding antibodies were just above the limits of detection (Supplementary Fig. S1). The same was shown for the levels of antibody classes of the animals exposed to PBS (control) nebulization (Fig. 1). In contrast, a significant increase was observed for both IgG_1_ and IgE levels when sensitized with aerosolic OVA. This was true for both the overall levels and the OVA-specific levels. When compared to controls, the TdT^−/−^ mice demonstrated a significant increase of all analyzed antibody classes (p < 0.0001). The same tendency was observed for the increase in antibody concentrations due to sensitization to the wild type, (total IgG_1_ p < 0.001, OVA-specific IgG_1_, total IgE and OVA-specific IgE p < 0.0001). However, in contrast to OVA-specific antibody levels, total IgE levels in the TdT^−/−^ mice were significantly lower than in wt (p < 0.0001).

### T_H_2 cytokines in the bronchoalveolar lavage are reduced in TdT^−/−^ mice

**Figure 2:**
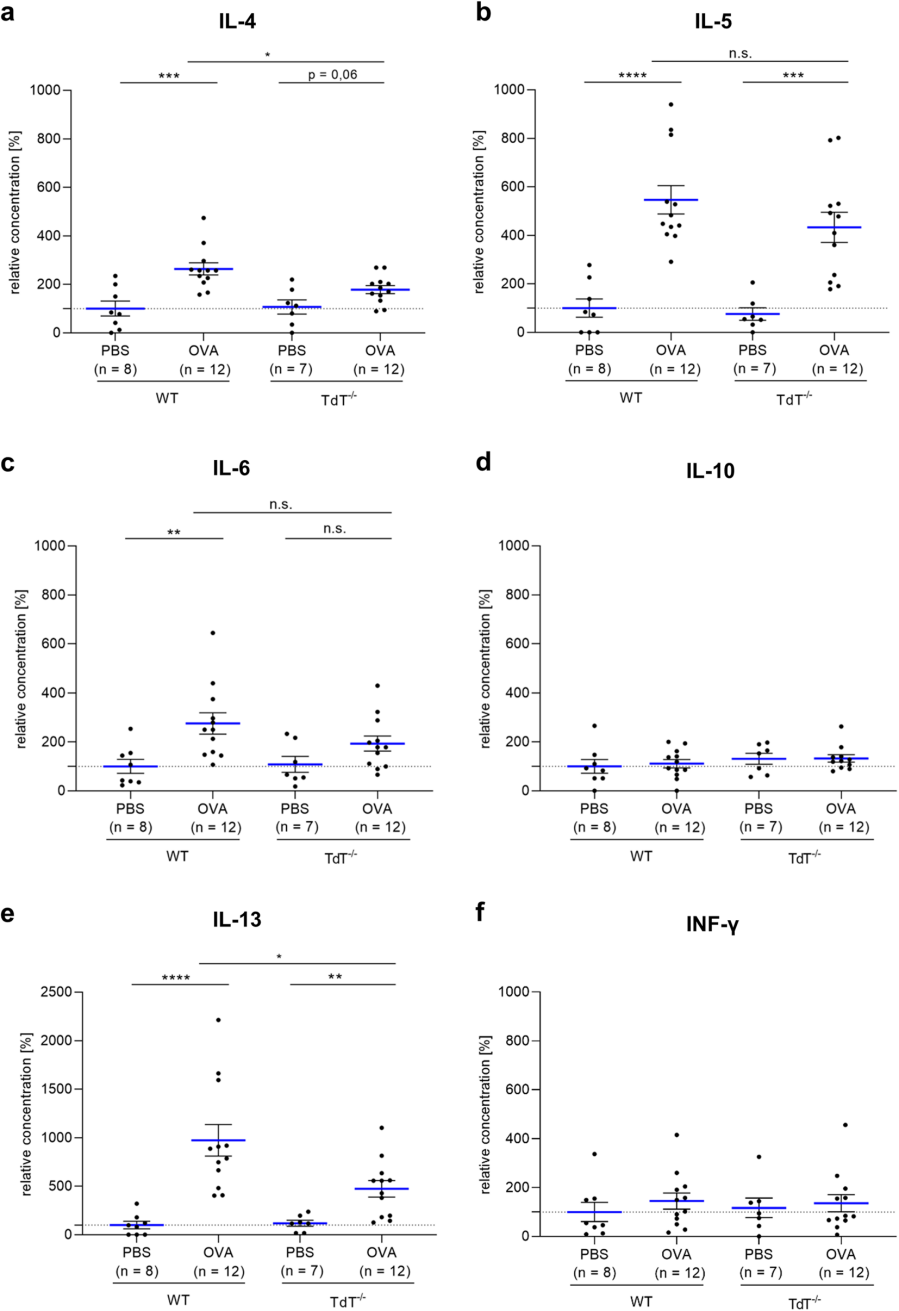
Cytokine level in BAL fluids. In BAL fluids of *wt* and TdT^−/−^ mice, both sensitized and non-sensitized, cytokine levels of (**a**) IL-4, (**b**) IL-5, (**c**) IL-6 (**d**) IL-10 (**e**) IL-13 and (**f**) INF-γ were measured using Cytokine Multiplex Assay. (**a**–**c**) Sensitization with OVA resulted in a marked increase in cytokine levels of IL-4 in TdT^−/−^ (p = 0.06) compared to non-sensitized mice. A significant rise was observed in IL-4 levels in *wt* (p < 0.001), in IL-13 (*Wt* p < 0.0001; TdT^−/−^ p < 0.01) and IL-5 (*Wt* p < 0.0001; TdT^−/−^ p < 0.001) compared to non-sensitized mice. However, this increase was attenuated in sensitized TdT^−/−^ mice compared to *wt* mice (p < 0.05 (IL13 and IL-4)). (**d**–**f**) The levels of the cytokines IL-6, IL-10 and INF-γ showed no differences between the two genotypes. All levels were normalized to *wt* control (mean shown as blue lines, SEM shown as black bars).

The local T_H_ cytokine pattern in BAL fluids of the animals was characterized by using a cytometric bead assay to determine IL-4, IL-5, IL-6, IL-10, IL-13 and interferon-γ (INF-γ) levels (Fig. 2). When compared to non-sensitized mice, sensitization with OVA followed by local allergen challenge resulted in a significant rise in IL-4 levels in wt mice (p < 0.001, 263.9 ± 25%; mean ± SEM) and a marked increase in TdT^−/−^ mice (p = 0.06, 178.3 ± 16.7%; mean ± SEM) in BAL fluids. The same tendency was observed for IL-13 levels (*wt* p < 0.0001, 972.4 ± 162.5%; TdT^−/−^ p < 0.01, 473.3 ± 85.7; mean ± SEM) and IL-5 (wt p < 0.0001, 546.5 ± 58.7%; TdT^−/−^ p < 0.001, 433.2 ± 62.0; mean ± SEM). However, when compared to wt mice, this increase was attenuated in sensitized TdT^−/−^ mice for IL-13 and for IL-4 (p < 0.05). The induction of IL-5 levels following OVA sensitization was comparable between the two genotypes. Sensitization with OVA did not lead to a significant increase in either IL-10, INF-γ or IL-6 levels in TdT^−/−^ mice, while IL-6 levels were significantly elevated after sensitization in wt (p < 0.01). The measured levels of the cytokines IL-6, IL-10 and INF-γ showed no differences between the two genotypes.

### Eosinophilic influx into the airways is reduced in TdT^−/−^ mice

**Figure 3:**
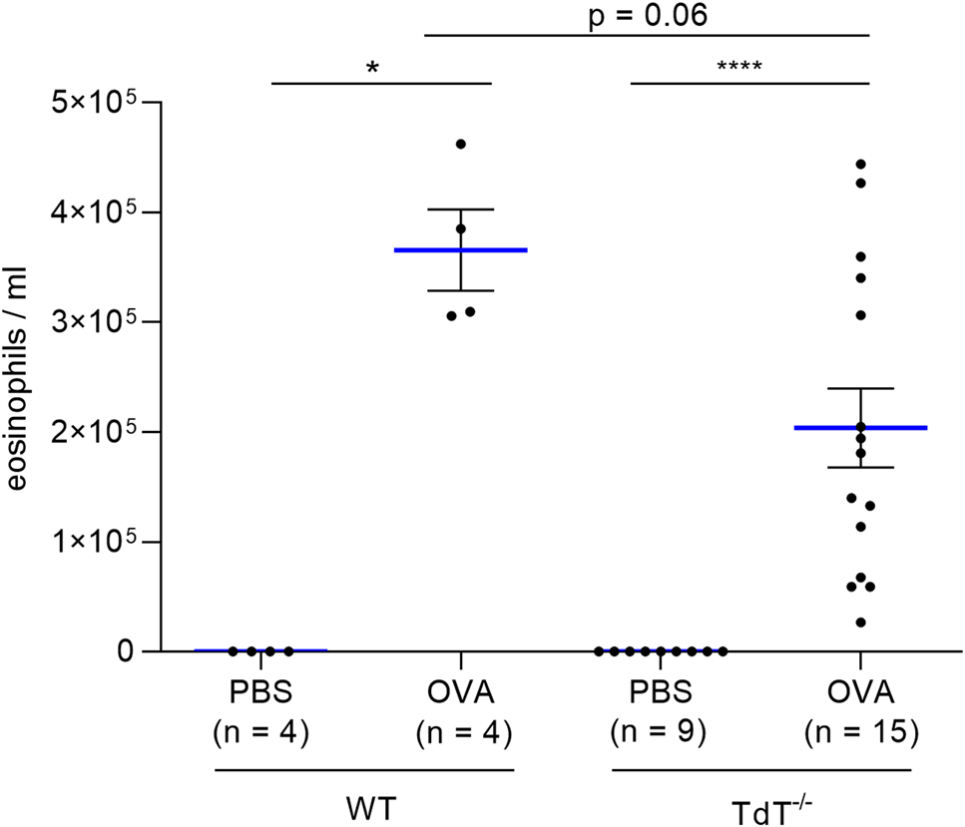
Content of eosinophils in bronchoalveolar lavage (BAL) fluids. In BAL fluids of *wt* and TdT^−/−^ mice, both sensitized and non-sensitized, content of eosinophils were measured using a pulse area counter. No eosinophils were detectable in the BAL fluids of non-sensitized animals. This applies to both genotypes, *wt* and TdT^−/−^. Sensitization and aerosolic challenge with OVA caused a significant influx of eosinophils (*wt* p < 0.001 and TdT^−/−^ p < 0.0001) in both genotypes. However, this influx was markly reduced in TdT^−/−^ mice compared to *wt* mice (p = 0.06; mean shown as blue lines, SEM shown as black bars).

Eosinophilic infiltration is associated with the development of local allergic airway inflammation. Thus, to assess the local inflammatory response in the airways, the influx of eosinophilic granulocytes into the BAL fluids was determined (Fig. 3). The number of eosinophils in BAL fluids in non-sensitized mice of either genotype proved negligible. As expected, sensitization followed by aerosolic challenge with OVA resulted in a significant increase in the levels of eosinophils in the BAL fluids of both wt (p < 0.05, 3.66 × 10^5^ ± 0.37 × 10^5^ cells/mL; mean ± SEM) and TdT^−/−^ mice (p < 0.0001, 2.04 × 10^5^ ± 0.36 × 10^5^ cells/mL; mean ± SEM). However, when compared to wt, the eosinophilic influx was markly reduced in the TdT^−/−^ animals (p = 0.06).

### Airway hyperresponsiveness is unset in TdT^−/−^ mice

**Figure 4:**
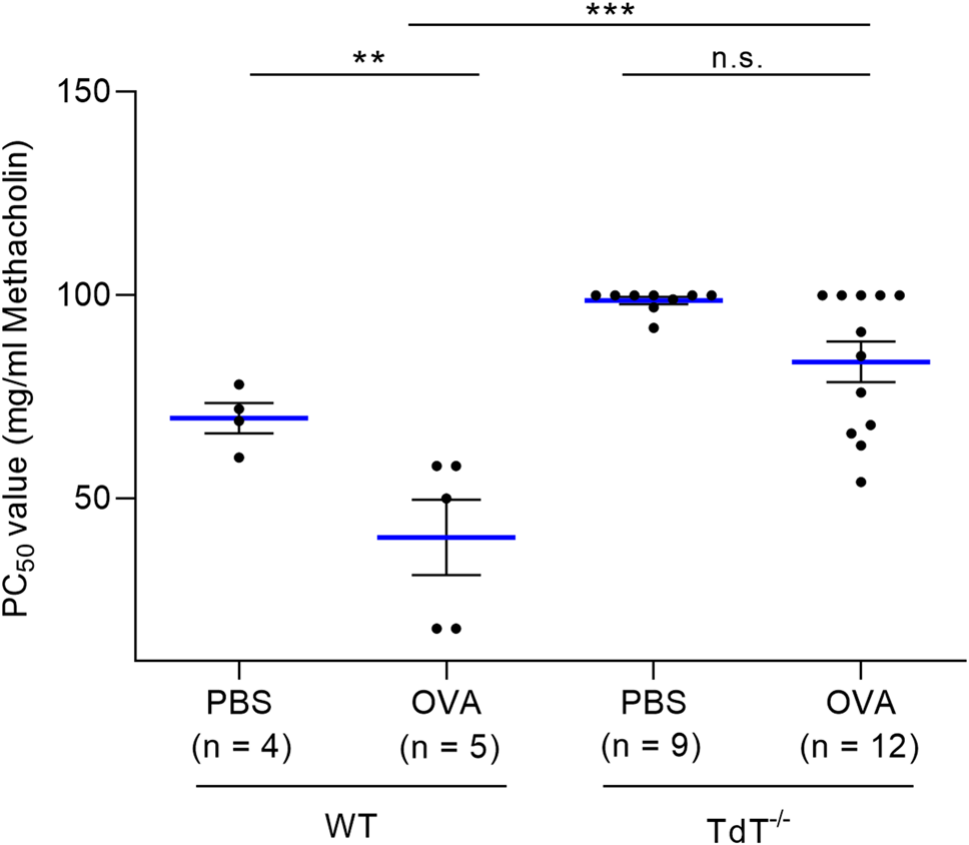
Lung function analysis—methacholine response. Head-out body plethysmography was used to assess lung function on day 29. Methacholine provocation was performed in the headout body plethysmograph to evaluate airway responsiveness. The PC_50_ value represents the provocation concentration of methacholine required to induce significant airway obstruction. In *wt* animals, PC_50_ was significantly reduced in the group of OVA-sensitized and challenged mice compared to the PBS control (p < 0.01). In TdT^−/−^ mice, no significant difference was found between OVA-sensitized animals and the control, thus airway hyperreactivity was attenuated (mean shown as blue lines, SEM shown as black bars).

To assess airway hyperresponsiveness, the response to inhaled methacholine was investigated (Fig. 4). The provocation concentration 50 (PC_50_) value was determined for statistical comparison of the individual groups. This value describes the concentration of methacholine (in mg/ml) that leads to a 50% decrease in the MEF_50_ value. For the wt animals, PC_50_ was significantly reduced in the group of OVA sensitized and challenged mice compared to the PBS control (p < 0.01). There is considerable indication that the degree of airway reactivity differed between the two genotypes, as in TdT^−/−^ mice, PC50 values were indistinguishable between sensitized and non-sensitized animals. Thus, OVA-induced airway hyperreactivity was abolished in the TdT^−/−^ group.

## Discussion

In a mouse model of experimental allergic inflammation, we found that CDR3 repertoires somatically diversified by N nucleotide addition are required for the development of a fully established allergic airway inflammation.

During ontogeny, as new cell types appear and tissues and organs are created, the developing humoral immune system encounters a progressively increasingly complex array of self-antigens. Sequential exposure to these normal ‘neo’antigens poses a risk for the development and survival of potentially pathogenic autoreactive immunoglobulins. The evolutionary adaptation in mammals that allows implantation of their embryos in the mother's womb creates an additional immunological problem^22^. Intimate contact with the mother's uterine tissue and leakage between the fetal and maternal circulations make maternal cells, tissues and organs yet another potential target for the developing immune system of the embryo and fetus. The infant inherits only half of maternal gene polymorphisms, thus the developing embryo and fetus can be considered a 'semi-allograft', with the potential for an attack on the mother by antibodies generated by her child.

Unsurprisingly, there is strong evidence that humoral immune responses in the mammalian embryo and fetus are selectively suppressed. This was made evident more than 40 years ago when the existence of a homologous, controlled, programmed hierarchy of antigen responsiveness was identified in lambs, mice, and humans^23,24^. This finding appeared paradoxical given that lymphocyte antigen receptor repertoires, immunoglobulin (Ig)^25^ and T cell receptor (TCR)^26^, were presumed to be generated in a stochastic fashion through the process of random VDJ rearrangement and N addition. However, it was subsequently shown that both the process of VDJ rearrangement and the presence and extent of N addition are regulated during embryonic, fetal and neonatal development, restricting the diversity of both repertoires in the womb.

The focus of antigen receptor diversity is the third complementary determining region of the V domain (CDR3), which is somatically created by V(D)J rearrangement and N nucleotide addition. CDR3 regions are located at the center of the antigen binding site, as classically defined, and thus typically play a commanding role in antigen recognition and binding^27^. Although restriction of antigen receptor CDR3 repertoires is common during mammalian ontogeny, the precise array and combination of mechanisms used vary by species. In mouse, the primary mechanism of repertoire control is the absence of terminal deoxynucleotidyl transferase (TdT) activity, the source of N nucleotide addition, until after birth, which restricts the diversity of both the Ig heavy (H) chain^28,29^ and TCRβ^13^ repertoires to germline content. Use of individual VH and, to a lesser extent, DH are also regulated^30^.

We and others have tested for benefits and detriments to lymphocyte development and immune function in TDT deficiency. The primary benefit to the absence of N nucleotides, and thus a focus on a germline-encoded TCR and Ig repertoire, is enhanced efficiency of positive selection and a more rapid population of lymphoid organs^31,32^.

With two exceptions, the effect on antibody production, T cell function, specificity, and pathogen neutralization is either neutral (e.g. ovalbumin KLH)^33^ or negative^34^. In T cells, the peripheral repertoire is more polyreactive and less peptide-oriented than is the N+ repertoire^31^. Total antibody production to a large array of antigens is neutral or reduced^32^. N nucleotide deficient CD8+ memory T cells mediate poor recall responses compared to adults and are comprised of a repertoire of lower avidity^35,36^. Responses towards some epitopes are skewed^36^, and heterosubtypic immunity to influenza virus is abrogated^34^. This manuscript was focused on testing one additional gap in our knowledge, i.e. the contribution of N nucleotide diversity to CD4 T cell mediated immune responses in general, and to allergens in specific.

Here we demonstrate a role of N addition in the function of a T_H_2 CD4 T cell-dependent IgE immune response to an allergen. We observe that the absence of N nucleotides leads to a complex disturbance of an allergen-induced inflammatory network, indicating a differentially altered sensitization phase and effector phase of this CD4 T cell dependent allergic response. The initial sensitization with ovalbumin using Alum as an adjuvant specifically led to an altered TdT deficient T_H_2 type antibody response since total IgG_1_ levels were increased whereas total IgE levels were decreased when compared to wt mice.

Allergen-specific IgE levels proved similar in TdT^−/−^ and wildtype mice indicating that the absence of N nucleotides did not entirely impair the absolute quantity of specific antibody production. This finding is in accordance with previous reports on other antigens^21,31,37–39^. However, the T_H_2 cytokines IL-4 and IL-13 were reduced in the BAL fluids of TdT^−/−^ mice, indicating that at least part of the problem reflected an effect of the altered repertoire on the intrinsic function of CD4 T cells, themselves, potentially due to the skewing of epitope recognition that occurs in the absence of N addition^36^. Unexpectedly, the allergic inflammation appeared to be reduced in the TdT^−/−^ mice in comparison to wt, as shown by reduced influx of eosinophils and an attenuated bronchial hyperresponsiveness to methacholine. However, head-out body plethysmography must be interpreted cautiously because of the difference in sample numbers, and it remains unclear whether the apparent difference between the wt and TdT^−/−^ control groups reflects an intrinsic difference in airway response between the two genotypes or experimental variance.

Increased total IgG_1_ levels and reduced total IgE levels indicate that in TdT^−/−^ mice the cytokine milieu contains more T_H_1 components than in wt controls. The weakened T_H_2 cytokine bias might result from a disruption of the positive feedback loop that usually links the sensitization phase that yields specific IgE under the influence of T_H_2 cytokines and the effector phase that follows re-exposure to the allergen and T_H_2 cytokine release by mast cells and basophils^40–45^, potentially due to changes in epitope recognition patterns. Based on the results of this study, future investigations should be conducted to provide better insight into the underlying mechanisms.

The polyreactivity of the B-cell repertoire of TdT^−/−^ mice is reduced compared to *wt* animals^38^. Thus, it remains unclear if the allergen specific IgE antibodies expressed by TdT^−/−^ mice have the same affinity as those of wildtype mice. Just like T cell patterns of epitope recognition, it can be speculated that specific IgE produced by TdT^−/−^ mice might not recognize the complete range of epitopes of ovalbumin bound by wild type IgE, thereby narrowing its reactivity to the allergen^46^. CDR-H3 length has significant influence on the tertiary structure of the antigen binding site^47^ and the length of CDR-H3 regions correlates with the nature of recognized antigens^48^. Thus TdT^−/−^ mice might be able to produce a similar quantity of allergen specific IgE as wildtype mice, but a broader, wildtype spectrum of allergen specific antibodies might be required to yield a clinically relevant allergic airway response. This would support the hypothesis that allergies may represent a misled oligoclonal allergen-specific immune response.

The OVA-induced airway hyperresponsiveness to methacholine ws reduced in TdT^−/−^ mice when compared to wild-type mice. The expression of IL-5 is normally triggered by elevated levels of IL-4, IL-13 and IgE. Intriguingly, the concentration of IL-5 was not reduced in BAL fluids of TdT^−/−^ mice. These observations support the hypothesis that the initial IgE-mediated inflammation was triggered without T-cell stimulation. Allergic inflammation does not exclusively depend on a T_H_2-dependent, IgE-mediated allergic type 1 immune response. Other factors, such as innate lymphoid cells (ILC2s), can also contribute by secreting IL-5 and IL-13, which in turn contribute to the adaptive type 2 immune response^49–52^. Since the concentration of IL-5 in BAL fluids in TdT^−/−^ mice was similar to wildtype, but the number of eosinophils tend to be reduced, mediators other than IL-5 might be involved in regulating the influx of eosinophils into the BAL fluids^53,54^. We favor the view that the reduced number of eosinophils in the BAL fluids can be attributed to the disturbed overall cytokine profile in TdT^−/−^ mice.

Although the diversity of both the Ig and TCR repertoire is restricted during ontogeny in both human and mouse, the precise timing and details differ^55^. The range of CDR-H3 lengths in humans is much larger compared to mice, although the murine CDR-H3 repertoire does not represent a subset of that of humans^56^. Moreover, in mouse the Ig and TCR repertoires remain deficient in N addition until days after birth; whereas in human N addition is limited in the embryo, begins to increase in the fetus in the second trimester of pregnancy, and achieves an adult phenotype by 6 months after birth^57^. Thus, it is unclear if these results can be transferred to human on a one-for-one basis. It also needs to be taken into account that these results might not necessarily reflect all type one allergies other than OVA. However, recent studies have shown that protection against allergy may begin in the womb, with maternal IgG being transferred to the embryo from the second trimester on, offering protection against allergen sensitization^58^. Thus, it is possible that exposure to allergens in the human womb in mothers who lack protective IgG may promote expression of a ‘locked-in’ allergen-sensitive Ig or TCR repertoire that will result in an increased risk of allergy after birth. Consequently, the differences in repertoire pre and post birth could lead to markedly different outcomes.

Studies of T cell function in the absence of TdT have either been general (e.g.^33^, or focused on CD8 T cell function^35,36,59,60^. Our findings suggest that equal attention should be placed on the study of the effects of the role of N addition in CD4 T cell epitope recognition and function^61^.

The mucosa associated lymphatic tissue is a regulator of the adaptive immune response and closely interacts with intestinal microbiota which are considered key regulators of health and disease^62–64^. To date, to the best of our knowledge, the microbiome of TdT^−/−^ mice has not been characterized in detail. However, the microbiome of RAG deficient mice that are unable to perform somatic recombination of the immunoglobulin heavy an light chains, is severely biased^65–69^. Thus, it is likely that TdT^−/−^ mice also exhibit an altered microbiome. It can be speculated that an altered microbiome may have an influence on the establishment of the allergic airway inflammation in TdT^−/−^ mice.

This study was able to shed light on just the section of the complex immune network that depends on the activity of TdT. The results that we obtained indicate that the absence of TdT leads to an alteration in the pattern of cytokine production after allergic sensitization and challenge; a feature that had not been described previously. Further studies are therefore necessary in order to present a detailed mechanism in this context. To identify further TdT-affected key components of the immune system, -omics experiments should be considered. In addition, it is necessary to investigate which specific genetic changes concerning the TCR might lead to a rescue of the TdT phenotype.

In conclusion, we found indications that the allergic phenotype is attenuated in a murine model of allergic airway inflammation in mice expressing “fetal-like” Ig and TCR repertoires, as reflected by the absence of N nucleotides. We hypothesize that a type 1 allergy is not only mediated by the antibodies specifically reacting in the ELISA, but properties of the N deficient TCR repertoire may also be involved in establishing and maintaining a normal T_H_2 cytokine profile that leads to the influx of eosinophils and airway hyperreactivity.

## Material and methods

### Study approval

The study was ethically approved by the governmental authority (Regierungspräsidium Giessen, Dezernat V54-Veterinärwesen; reference V54-19c20-15 (1) MR36-Nr. 05/2007). All procedures were performed in accordance with relevant guidelines and regulations.

### Mice

We have used a previously described TdT^−/−^ mouse strain on a BALB/c background^21,31^ using wildtype mice as controls (Harlan Winkelmann (Borchen, Germany)). All animals were kept under pathogen-free conditions in single ventilated cage systems. At a constant temperature of 20 °C, the mice were exposed to an artificial light–dark rhythm of 12/12 h. The animals were offered an ovalbumin-free diet and water ad libitum for food and drinking water intake. The study was carried out in compliance with ARRIVE guidelines.

### Test protocol

**Figure 5:**
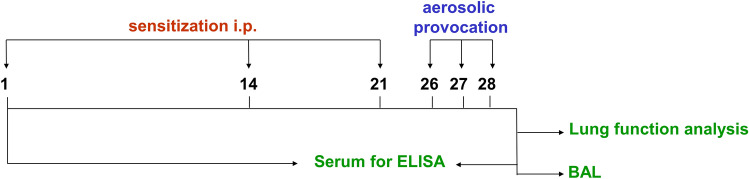
Test protocol. Mice were sensitized to ovalbumin (OVA) by intraperitoneal injections on days 1, 14, and 21. To induce an allergic airway inflammation, mice received allergen challenges via the airways delivered by nebulization with OVA on days 26, 27 and 28. The control group received PBS instead of OVA. To quantify the immunoglobulin classes in serum using ELISA, blood samples were taken on the day before the first sensitization and on day 30. Head-out body plethysmography including methacholine provocation was used to assess the lung function on day 29. Bronchoalveolar lavage was performed on day 30. BAL fluids were evaluated for cytokine levels as well as eosinophil content.

The chronological sequence of operations and analyses was designed as follows (Fig. 5). Before starting of the test series, the concentration of individual antibody classes was measured in serum by ELISA. On days 1, 14, and 21, the mice were intraperitoneally sensitized with either the allergen ovalbumin (OVA) or with PBS as a control. On days 26, 27, and 28, the aerosol provocation was again performed with either OVA or PBS. The resulting effects on serum antibody concentration were subsequently determined by ELISA. In addition, lung function was examined. Bronchoalveolar lavage (BAL) was performed to assess both the number of eosinophils and the amount of characteristic cytokines in the BAL fluids.

### Protocol of allergic sensitization

The study included four groups of mice: (A) nonsensitized *wt* mice (*Wt* PBS), (B) sensitized *wt* mice (*Wt* OVA), (C) nonsensitized TdT^−/−^ mice, and (D) sensitized TdT^−/−^ mice. Mice were sensitized to ovalbumin (OVA) as previously described^8,20^. 10 µg of OVA grade IV (Sigma, Germany) were adsorbed to 1.5 mg Al(OH)_3_ (Imject^®^ Alum; Pierce, Rockford, Ill., USA) and administered by intraperitoneal injections on days 1, 14, and 21. To induce an allergic airway inflammation, mice received allergen challenges via the airways delivered by nebulization of 1% (w/v) OVA grade V (Sigma), diluted in PBS, for 20 min on days 26, 27, and 28. Control mice received PBS intraperitoneally by intraperitoneal injections on days 1, 14, and 21 and were challenged on days 26, 27, and 28 with aerosolic PBS (Table 1).

**Table 1:** Model of allergic airway inflammation.

Genotype	Sensitization	Injection (i.p.)	Aerosolic application
TdT^−/−^	Sensitized	OVA IV	OVA V
	Non-sensitized	PBS	PBS
*wt* (BALB/c)	Sensitized	OVA IV	OVA V
	Non-sensitized	PBS	PBS

### Determination of antibody titers

To quantify the immunoglobulin levels in serum, blood samples were taken on the day before the first sensitization and on day 30. Serum concentrations of total and allergen-specific IgE and IgG_1_ were measured by ELISA as previously described^20^. Antibodies and standards were purchased from BD (Heidelberg, Germany). Serum samples were diluted 1:500 (IgG_1_) and 1:100,000 (IgE) for OVA sensitized mice. For non-sensitized controls, samples were diluted 1:10 (IgG_1_) and 1:2 (IgE), respectively. Determination of OVA-specific IgE was performed as described in^8^.

### Assessment of lung function

As previously described, head-out body plethysmography was used to assess lung function on day 29^40^. Methacholine provocation was performed in the headout body plethysmograph to evaluate airway responsiveness (AR). The animals were not anaesthesized during the determination of lung function. After a period of acclimatization and recording of the baseline, the mice inhaled aerosol PBS. Methacholine was then applied in ascending concentration (6.25–100 mg/mL). The mean expiratory flow at which 50% of the tidal volume in ml/s has been exhaled (MEF_50_) was used to assess airflow limitation. The PC_50_ value represents the provocation concentration of methacholine required to induce significant airway obstruction.

### Bronchoalveaolar lavage

On day 30, bronchoalveaolar lavage was performed as described previously^8^: the trachea was cannulated and the airways were lavaged with 1.6 mL ice-cold PBS supplemented with proteinase inhibitors (Complete^®^; Boehringer, Mannheim Germany). Using a pulse area counter (Casy^®^, Schärfe, Reutlingen, Deutschland), the cell numbers were ascertained. Cells were centrifugated onto slides, differentially stained with DiffQuik^®^ (Behring, Marburg, Germany), and classified by light microscopy.

### Determination of cytokine levels in bronchoalveolar lavage fluids

In the BAL fluids, the concentration of interleukin 4 (IL-4), IL-5, IL-6, IL-10, IL-13, and interferon-γ (INF-γ) was determined. For the analysis, the Basic Kit FlowCytomix for mouse/rat (Bender Medsystems; Vienna, Austria) was utilized according to manufacturer's specifications using a FACSCalibur FlowCytometer (BD, Franklin Lakes, USA).

### Statistical analysis

Data are presented as mean ± SEM. The two‐tailed Mann–Whitney–U-test was used to determine statistical significance. *p* < 0.05 was considered statistically significant. For labelling in illustrations, the following symbols are used: *p < 0.05; **p < 0.01; ***p < 0.001; ****0.0001. Statistics were performed using GraphPad Prism software V8.02 (GraphPad Software, La Jolla, CA, USA).

## Supplementary Information

Supplementary Fig. S1.

## Abbreviations

BAL: Bronchoalveolar lavage
CD: Cluster of differentiation
CDR3: Complementarity determining region 3
CDR-H3: Immunoglobulin heavy-chain complementarity-determining region 3
D_H_: Diversity
Ig: Immunoglobulin
IL: Interleukin
ILC2: Innate lymphoid cell
MEF: Mean expiratory flow
OVA: Ovalbumin
PBS: Phosphate buffered saline
PC: Provocation concentration
SEM: Standard error of the mean
TCR: T cell receptor
TdT: Terminal deoxyribonucleotidyl transferase
Wt: Wild type
