# Supplementary material for: Attenuated asthma phenotype in mice with a fetal-like antigen receptor repertoire
Source: Sci Rep. 2021 Jul 9;11:14199. doi: 10.1038/s41598-021-93553-6 (PMC8270943; doi:10.1038/s41598-021-93553-6)

Supplementary file

Attenuated asthma phenotype in mice with a fetal-like antigen receptor repertoires

Regine Stutz^1^, Christopher Meyer^1^, Elisabeth Kaiser^1^, Sybelle Goedicke-Fritz^1,2^, Harry W Schroeder Jr^3^, Robert Bals^4^, Christoph Haertel^5^, Tobias Rogosch^2^, Sebastian Kerzel^2,6^, Michael Zemlin ^1,2,*^

^1^ Department of General Pediatrics and Neonatology, Saarland University Medical School, Homburg, Germany

^2^ Department of Pediatrics, Philipps-University Marburg, Marburg, Germany

^3^ Department of Medicine, University of Alabama at Birmingham, Birmingham, AL, United States

^4^ Department of Internal Medicine V - Pulmonology, Allergology and Critical Care Medicine, Saarland University, Saarland University Medical School, Homburg, Germany

^5^ Department of Pediatrics, Würzburg University Medical Center, Würzburg, Germany

^6^ Department of Pediatric Pneumology and Allergy, University Children's Hospital Regensburg, Campus St. Hedwig, Regensburg, Germany

***** Michael.zemlin@uks.eu

Supplementary Figure S1: Serum immunoglobulin levels before sensitization

In serum of *wt* and TdT^-/-^ mice before sensitization with OVA, immunoglobulin levels of (**a**) total IgG_1_, (**b**) total IgE, (**c**) OVA-specific IgG_1_ and (**d**) OVA-specific IgE were measured using ELISA. The measured levels of antibodies of all mouse strains were negligible. No significant differences were detected (mean shown as blue lines, SEM shown as black bars).


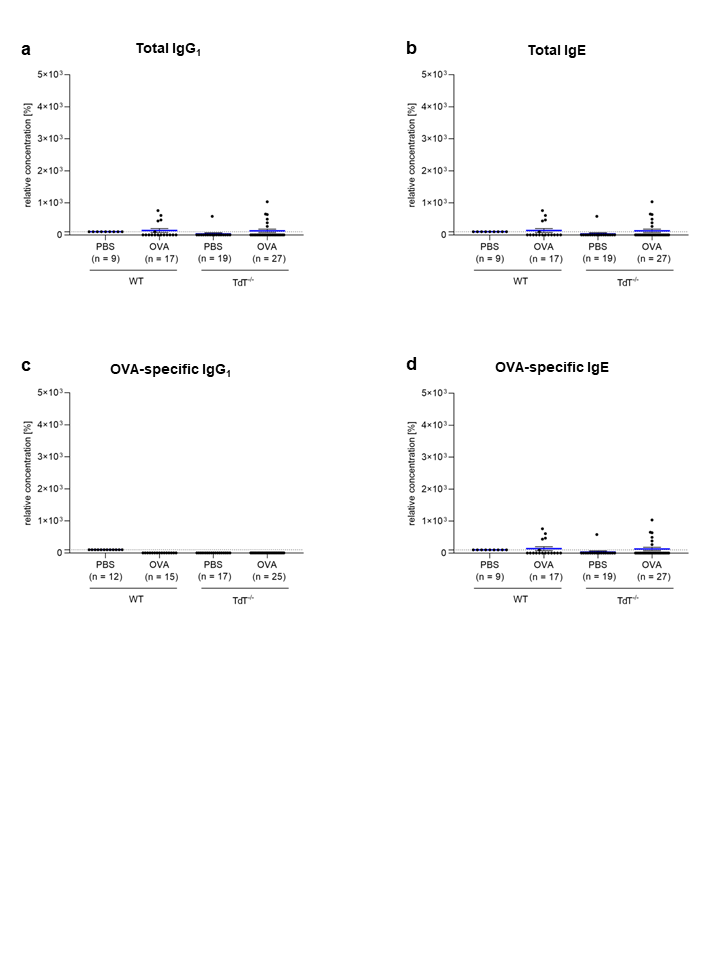

Supplement: Supplementary file 1 — Supplementary Fig. S1. [file 41598_2021_93553_MOESM1_ESM.docx]
